# Transmission traits of malaria parasites within the mosquito: Genetic variation, phenotypic plasticity, and consequences for control

**DOI:** 10.1111/eva.12571

**Published:** 2017-12-16

**Authors:** Thierry Lefevre, Johanna Ohm, Kounbobr R. Dabiré, Anna Cohuet, Marc Choisy, Matthew B. Thomas, Lauren Cator

**Affiliations:** ^1^ MIVEGEC, IRD, CNRS University of Montpellier Montpellier France; ^2^ Institut de Recherche en Sciences de la Santé (IRSS) Bobo Dioulasso Burkina Faso; ^3^ Laboratoire Mixte International sur les Vecteurs (LAMIVECT) Bobo Dioulasso Burkina Faso; ^4^ Department of Entomology and Center for Infectious Disease Dynamics Penn State University University Park PA USA; ^5^ Oxford University Clinical Research Unit Hanoi Vietnam; ^6^ Grand Challenges in Ecosystems and Environment Imperial College London Ascot UK

**Keywords:** host–parasite interactions, malaria, mosquito, transmission

## Abstract

Evaluating the risk of emergence and transmission of vector‐borne diseases requires knowledge of the genetic and environmental contributions to pathogen transmission traits. Compared to the significant effort devoted to understanding the biology of malaria transmission from vertebrate hosts to mosquito vectors, the strategies that malaria parasites have evolved to maximize transmission from vectors to vertebrate hosts have been largely overlooked. While determinants of infection success within the mosquito host have recently received attention, the causes of variability for other key transmission traits of malaria, namely the duration of parasite development and its virulence within the vector, as well as its ability to alter mosquito behavior, remain largely unknown. This important gap in our knowledge needs to be bridged in order to obtain an integrative view of the ecology and evolution of malaria transmission strategies. Associations between transmission traits also need to be characterized, as they trade‐offs and constraints could have important implications for understanding the evolution of parasite transmission. Finally, theoretical studies are required to evaluate how genetic and environmental influences on parasite transmission traits can shape malaria dynamics and evolution in response to disease control.

## 1 INTRODUCTION

Human malaria remains one of the most common causes of human mortality, accounting for nearly half a million deaths each year (WHO, 2015). Malaria is caused by *Plasmodium* parasites transmitted among humans by the bites of infected *Anopheles* mosquitoes (Box 1). More than 85% of malaria cases and 90% of malaria deaths occur in sub‐Saharan Africa, mainly among young children (WHO, 2015). Five species of the genus *Plasmodium* cause all human malaria infections. Of these parasites, *Plasmodium falciparum* causes the highest mortality and presents one of the most pressing challenges facing public health systems worldwide (White et al., 2014; WHO, 2015). Ongoing control efforts, relying mostly on antimalarial drugs and insecticide‐based interventions such as long‐lasting insecticidal nets and indoor residual spraying against mosquito vectors, have reduced malaria transmission (Bhatt et al., 2015). However, these interventions have selected for drug and insecticide resistance which could jeopardize control efforts (Huijben & Paaijmans, 2017; Sternberg & Thomas, 2017).

Box 1 **Malaria life cycle and the contrasting amount of knowledge on transmission traits variability within‐vertebrate hosts versus within‐mosquito vectors**

1

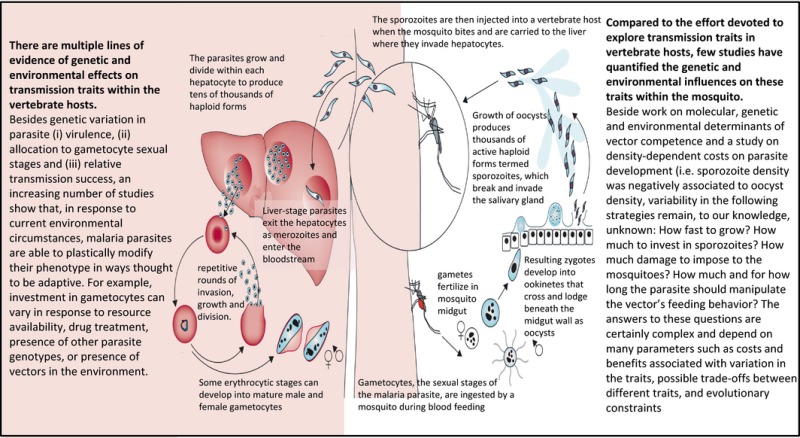

Despite the public health importance of these pathogens, many fundamental aspects of transmission remain unexplored. In particular, the sources of variation in traits that predict transmission from vectors to vertebrate hosts have been largely overlooked (Box 1). Like any vector‐borne parasite, malaria parasites must exploit patchy resources, encountering different environments with varying resources and selective forces as they make their way between the human host and insect vector. Parasite transmission traits can thus be influenced by multiple interacting factors including the direct influence of parasite genetic characteristics, the within‐vertebrate or within‐vector environment (vertebrate/vector genotype, immune responses, resource availability, presence of co‐infecting parasites, age, etc.), and the indirect influence of the external environment (temperature, humidity, host's predators, competitors, etc.). In recent years, a great deal of effort has been invested in studying transmission traits of malaria parasites in their vertebrate host (Cameron, Reece, Drew, Haydon, & Yates, 2013; Greischar, Mideo, Read, & Bjornstad, 2016; Neal & Schall, 2014; Reece, Ramiro, & Nussey, 2009). As we would predict, studies have shown that both genetic and environmental factors are important in determining parasite transmission from vertebrate hosts to mosquitoes. Like any other phenotypic trait, transmission traits can respond to environmental changes either plastically or evolutionarily (Box 2). For example, work using rodent malaria models suggests that parasite genotype can predict virulence and transmission success (De Roode et al., 2005). Furthermore, studies have shown that the investment of malaria parasites in gametocyte transmission stages can vary in response to environmental conditions, such as the presence of drugs, the availability of resources, the host immune response, coinfection with different strains, and the presence of vectors (Cornet, Nicot, Rivero, & Gandon, 2014; Mideo & Reece, 2012; Pollitt et al., 2011). While some of these responses may illustrate cases of passive susceptibility to environmental changes, others are likely examples of adaptive plasticity (Box 2). For example, *Plasmodium chabaudi* can detect the presence of unrelated conspecifics and adjust the proportion of male and female gametes in a way that supports sex ratio theory (Reece, Drew, & Gardner, 2008). This research demonstrates that unicellular parasites can evolve finely tuned mechanisms to detect information about their within‐host environment and plastically adjust some of their transmission traits.

Box 2 **Genetically fixed responses and (adaptive vs. nonadaptive) phenotypic plasticity**

1 Like any other organism trait, changes in parasite phenotypic traits can occur through two nonmutually exclusive processes: genetically fixed responses and/or phenotypic plasticity (Pigliucci, 2005). First, there may be genetic variation underlying transmission traits, and natural selection will favor the genetic variants which produce the phenotypes most fitted to the current conditions. This is the classic evolutionary response whereby some genetic variants can spread through the population over generations. Genetic variation is the raw material for evolution; therefore, characterizing genetic variability in transmission traits is key to understanding how control interventions can drive evolutionary changes in the parasite. As one hypothetical example, reduced vector longevity following insecticide exposure might select individuals with shorter EIP in the parasite population.

Second, a given parasite genotype may be able to produce different phenotypes in response to different environmental conditions, that is, phenotypic plasticity. In contrast to genetic changes over generations, modifications in phenotypic traits through plasticity can occur within a generation. Many examples of phenotypic plasticity are clearly adaptive such as some immune responses, antipredator defenses, and diapauses allowing individuals to adjust to environmental variation in real time (Whitman & Agrawal, 2009). In this case, organisms possess mechanisms to detect cues that predict environmental changes and induce adaptive plasticity. Such plasticity does not necessarily involve changes in gene frequencies in the parasite population and can provide a more rapid response to unpredictably changing environments. Using the above hypothetical example, parasites could detect cues associated with imminent death of their vectors (e.g., directly through the presence of insecticides or indirectly through modifications of vector physiology) and adaptively accelerate their sporogonic development to achieve transmission prior to vector death.

In contrast to adaptive plasticity, other environmentally induced changes in phenotype may illustrate mere susceptibilities to environmental stresses with no adaptive value (Ghalambor, McKay, Carroll, & Reznick, 2007). In this case, the phenotypic changes can arise from a “passive” disruption of physiological processes and do not require any mechanisms for how cues are detected. For example, a longer EIP in mosquitoes exposed to insecticides and hence with reduced potential for transmission compared to mosquitoes with greater longevity would indicate that environmental variation (here a reduction in mosquito longevity) does influence this trait, but this would also be intuitively interpreted as a case of phenotypic plasticity with maladaptive value. However, it is often difficult to conclude whether or not altered phenotypes are adaptive or nonadaptive (Pigliucci, 2005).

In any case, determining the extent to which parasite transmission traits are genetically fixed or plastic will help predict the consequences of control interventions on parasite evolution. Experimental designs with some form of genetic structure (clones, family lines) and environmental treatments are extremely powerful for studying genetic effects and phenotypic plasticity (Whitman & Agrawal, 2009). Measuring transmission trait (EIP, virulence, manipulation, infection level) variation among different genetic backgrounds or environmental conditions will help to quantify the relative importance of phenotypic plasticity and genetic variation. The statistical measure of variation is variance, which quantifies the deviation of values around a mean. The variance of a phenotypic trait can be partitioned as follows:

*V*_*P*_ **= *V*_*G*_ **+ *V*_*E*_ **+ *V*_*G*×*E*_ **+ *V*_error_

where *V*_*P*_ **= Total phenotypic variance for a trait;*V*_*G*_ **= Genetic variance (proportion of phenotypic variation attributable to genes);*V*_*E*_ **= Environmental variance (proportion of variation caused by the environment);*V*_*G*×*E*_ **= Genotype × Environment interaction (genetic variation for phenotypic plasticity);*V*_error_ = Unexplained variance, including developmental noise.

Quantifying phenotypic variation across different parasite clones or mosquito genotypes in controlled conditions will minimize environmental variance, and the phenotypic variance will be close to the genetic variance. Similarly, randomly assigning mosquito genotypes infected with single parasite clones (monoclonal infections) to different environmental treatments will lead to a robust estimate of phenotypic plasticity (Whitman & Agrawal, 2009).

In comparison with explorations of within‐host factors that affect transmission from hosts to vectors, little work has been performed on the other half of the parasite transmission cycle: from vectors to vertebrate hosts. We propose that a complete understanding of factors that shape the evolution of transmission strategies must consider not only the within‐vertebrate host factors contributing to transmission, but also those factors within the vector (Box 1). We use vectorial capacity (*C*), one of the most common metrics of transmission for vector‐borne diseases, to establish a framework for investigating genetic and environmental variation in transmission traits within the mosquito vector. *C* is defined as the potential intensity of vertebrate‐to‐vertebrate parasite transmission by mosquito vectors and can be described by the formula:

$$C=\frac{ma^{2}Vp^{n}}{-\ln(p)}$$

where *m* is the density of vectors per vertebrate hosts, *a* is the vector biting rate and host preference, *V* is vector competence, *p* is the daily probability of adult vector survival, and *n* is the duration in days of the parasite's extrinsic incubation period (EIP; Dye, 1992). Four of these critical components of transmission—the biting rate, mosquito competence, mosquito survival, and EIP—are traits that could potentially be determined directly or indirectly by parasites (Table 1). The vectorial capacity equation predicts that parasites could enhance transmission by influencing vector physiology to increase competence (*V*), altering the timing and propensity of mosquito biting (*a*), shortening EIP (*n*), or by increasing vector longevity (*p*).

**Table 1:** The critical components of malaria transmission that can either be determined directly or indirectly by parasites and how they affect our understanding of transmission

Component of vectorial capacity	Effect of increase in component on disease transmission (everything else being equal)	Interactions or trade‐offs to consider	Key questions to address	Applications and outlook
Mosquito competence (*V*)	↑	Virulence transmission trade‐off could result in mosquitoes with higher competence having reduced survival	Does mosquito competence correlates with *a*,* n* and/or *p*? Is competence predicted by *G*_*p*_ × *G*_*H*_ × *E*? Do environmentally driven changes in competence illustrate adaptive or nonadaptive phenotypic plasticity on the part of the parasite or the vector?	Understanding the genetic basis of competence can identify targets for genetic modification‐based control strategies
Vector biting rate and host preference (*a*)	↑	Biting rate increases mortality risk and could reduce vector survival. Changing host preference could increase survival by reducing exposure to insecticides	How does parasite impact vector biting rate? Can malaria parasites manipulate the vertebrate host choice of their vectors? Is malaria manipulation of vector biting rate a general phenomenon among the different mosquito–parasite combinations? Is there any parasite genetic variation for manipulation? Does the intensity of manipulation vary with environmental conditions (e.g., seasonally with mosquito densities)? Does *a* correlate with *n* and/or *p*?	Identifying parasite–mosquito associations that exhibit altered feeding behavior during infection will help improve transmission predictions by more accurately estimating biting rates and could also provide the opportunity to selectively target infected mosquitoes for control
The extrinsic incubation period (*n*)	↓	Faster developing parasites might inflict higher fitness costs on mosquitoes and reduce their ability to transmit	How does EIP length respond to within‐vector and environmental conditions? Can EIP be predicted by vector or parasite genotype? What affects EIP length besides temperature?	Shorter EIPs could evolve in response to interventions if there is a genetic basis for EIP length and sufficient selection pressure. For example, insecticides that reduce vector lifespan may favor faster parasite development
Mosquito longevity (*p*)	↑	Longer‐lived mosquitoes may have reduced biting rates	Do parasite traits, such as EIP or virulence, covary with mosquito lifespan? How does malaria infection impact vector lifespan? How does vector lifespan affect selection on parasites with different traits?	Mosquito longevity is the most sensitive parameter in vectorial capacity, and understanding how this trait covaries with other vector and parasite traits related to transmission is crucial for better characterizing transmission in the field

The degree to which variation in any one of these parameters affects transmission outcomes depends both on how sensitive vectorial capacity is to perturbations in a given parameter and the extent to which a given parameter can vary. Sensitivity analyses can evaluate the relative effect small changes in one parameter have on the outcome of what the model is predicting. Previous sensitivity analyses on the vectorial capacity equation have indicated that vectorial capacity is highly sensitive to adult mosquito survival (Brady et al., 2016; MacDonald, 1957; Smith & McKenzie, 2004). This has consequently led to suggestions that interventions targeting adult survival may be the most effective means of vector control, even when weighted by the relative effort of implementing an intervention (Brady et al., 2016). Using similar analyses to weight sensitivity by the capacity of a trait to vary cannot currently be conducted on key vector traits (*V*,* a*,* n*, and *p*) because variation in traits is poorly characterized. Control strategy design and transmission predictions could be improved by understanding the extent of variation in these parameters. Here, we explore each of these traits, review the extent of observed and predicted genetic and environmental variation, and discuss how variation in any one of these components of vectorial capacity impacts parasite transmission.

## 2 MOSQUITO COMPETENCE (*V*)

Mosquito competence is the ability of mosquitoes to support malaria development and transmission. It can be measured in the laboratory by exposing mosquitoes to a given dose of parasite gametocytes during blood feeding directly on an infected vertebrate host (Direct Feeding Assay (Bousema et al., 2012)), or through a membrane containing either cultured parasites (Standard Membrane Feeding Assays (van der Kolk et al., 2005)) or blood drawn from naturally infected hosts (Direct Membrane Feeding Assays (Bousema et al., 2012; Ouédraogo et al., 2013)). The measure of competence captures both parasite prevalence (the proportion of malaria‐exposed mosquitoes harboring at least one oocyst in their midgut or sporozoite in their salivary gland) and parasite intensity (the number of oocysts in the guts, or the number of sporozoites in the salivary glands of infected mosquitoes). Competence is a combined estimate of parasite infectivity (the parasite's ability to successfully establish and develop in the mosquito) and vector susceptibility to infection. It thus encompasses both mosquito resistance mechanisms used to fight the infection and parasite mechanisms used to overcome the vector's defenses.

The molecular and genetic bases of mosquito competence for malaria parasites have been well characterized for a number of mosquito–parasite associations (Aly, Vaughan, & Kappe, 2009; Beier, 1998; Bennink, Kiesow, & Pradel, 2016; Cirimotich, Dong, Garver, Sim, & Dimopoulos, 2010; Li et al., 2013; Molina‐Cruz et al., 2012; Redmond et al., 2015; Severo & Levashina, 2014; Sinden, 2016; Sinden, Alavi, & Raine, 2004). For example, different strains or families of *Anopheles gambiae*, the primary vector of malaria in Africa, display a wide range of susceptibility for a given parasite genotype (Blandin et al., 2009; Harris et al., 2010) and different *Plasmodium* isolates also vary in their infectivity to a given mosquito strain (Molina‐Cruz et al., 2012). Some studies have also demonstrated the existence of mosquito–parasite genetic interactions (Harris et al., 2012; Lambrechts, Halbert, Durand, Gouagna, & Koella, 2005; Molina‐Cruz et al., 2015). As yet, however, this large body of research has provided only limited insight into transmission dynamics in the field.

Besides mosquito and parasite genetic factors, there is a great diversity of ways in which biotic and abiotic external and within‐vector environmental factors (temperature, mosquito diet, insecticide exposure, microbial gut flora, infection history, mosquito age, etc.) can influence with mosquito competence (Alout, Djègbè, et al., 2014; Gendrin et al., 2015; Hien et al., 2016; Lefèvre, Vantaux, Dabiré, Mouline, & Cohuet, 2013; Murdock, Blanford, Luckhart, & Thomas, 2014; Murdock, Paaijmans, Cox‐foster, Read, & Thomas, 2012; Pigeault, Nicot, Gandon, & Rivero, 2015; Pollitt, Bram, Blanford, Jones, & Read, 2015; Shapiro, Murdock, Jacobs, Thomas, & Thomas, 2016; Takken et al., 2013; Vantaux, Dabiré, Cohuet, & Lefèvre, 2014). However, it is still unknown whether these environmentally driven changes in competence illustrate mere passive susceptibilities to environmental stresses (nonadaptive plasticity) or active beneficial shifts in either parasite growth and development or mosquito immune responses (parasite or vector adaptive phenotypic plasticity; Box 2).

Malaria transmission depends on the production of gametocytes that infect mosquitoes, which in turn develop in the mosquito vector to produce the transmissible stage of parasites, known as sporozoites (Box 1). Although there has been a great deal of effort to understand variation in gametocyte investment in several Plasmodium species (Bousema et al., 2008; Carter et al., 2013; Gadalla et al., 2016; McKenzie, Jeffery, & Collins, 2002; Neal & Schall, 2014; Box 1), it remains controversial as to whether or not the parasite is able to modulate its growth, survival, and sporozoite production within the mosquito vector. Similar to parasite stages within the vertebrate host, stages within the mosquito experience variation in their environment. Factors that may influence the parasite's within‐vector environment include vector age, resource availability, and presence of competitors. Whether the parasite is able to actively detect these variations and adjust its development through adaptive phenotypic plasticity remains enigmatic. In particular, it is still unclear whether intermediate “optimum” parasite densities exist for maximizing vector‐to‐vertebrate transmission. Parasite numbers during sporogonic development exhibit marked fluctuations, with the gametocyte to ookinete transition, the ookinete to oocyst transition, and the salivary gland invasion by sporozoites representing three major bottlenecks (reviewed in (Vaughan, 2007), see also Box 1). Studies using the *Plasmodium berghei—Anopheles stephensi* experimental system found that these developmental transitions experienced negative density dependence, possibly due to resources and space limitation and/or to an elevated mosquito immune response (Pollitt et al., 2013; Sinden et al., 2007). In addition, high‐density *P. berghei* infections can cause significant lifespan reduction in *An. stephensi* (Dawes, Churcher, Zhuang, Sinden, & Basanez, 2009; Pollitt et al., 2013). Together, the observations that high‐density infections limit both parasite development and vector survival support the possible existence of a selective pressure for parasites to modulate growth and reproduction within the vector to maintain densities at which transmission is maximized (Pollitt et al., 2013).

An important assumption of this hypothesis is that there must be a positive relationship between sporozoite burden in the salivary glands and infection of the vertebrate host, something that has long been disputed (Beier, Davis, Vaughan, Noden, & Beier, 1991; Beier et al., 1992; Ponnudurai, Lensen, Vangemert, Bolmer, & Meuwissen, 1991; Sinden, 2016). A recent study using rodent parasites provides strong support for this relationship by showing that mosquitoes with higher numbers of sporozoites in salivary glands are indeed more likely to transmit malaria (Churcher et al., 2017).

It has also been proposed that self‐restriction strategies based on programmed cell death may reduce the mosquito immune response, competition for resources, and/or increase vector survival, hence increasing parasite transmission probability (Al‐Olayan, Williams, & Hurd, 2002; Lüder, Campos‐Salinas, Gonzalez‐Rey, & van Zandbergen, 2010; Pollitt, Colegrave, Khan, Sajid, & Reece, 2010). However, suicide of some parasites may be beneficial only if this increases transmission of closely related individuals (i.e., increased indirect fitness) such as in monoclonal infection (Ameisen et al., 1995; Nedelcu, Driscoll, Durand, Herron, & Rashidi, 2011; Reece, Pollitt, Colegrave, & Gardner, 2011). This possible strategy has been supported by a number of observations showing that zygote and ookinete stages can indeed undergo apoptosis‐like processes (Ali, Al‐Olayan, Lewis, Matthews, & Hurd, 2010; Al‐Olayan et al., 2002; Arambage, Grant, Pardo, Ranford‐Cartwright, & Hurd, 2009; Pollitt et al., 2010). Further investigations are required to determine the extent to which the occurrence and intensity of parasite apoptosis depend on parasite / mosquito genotype and on the density and relatedness of co‐infecting parasites.

## 3 VECTOR BITING RATE AND HOST PREFERENCE (*a*): PARASITE MANIPULATION OF THE VECTOR'S FEEDING BEHAVIOR

The vectorial capacity equation predicts that, when ready to be transmitted from either vertebrate to vector or vector to vertebrate, malaria parasites able to increase the vector's biting rate on suitable vertebrate hosts species would increase their probability of transmission (Dobson, 1988). This “right bite at the right time” requirement of malaria transmission represents an extremely risky point in the parasite life cycle. Although evidence show that malaria parasites can enhance mosquito’s feeding rate (Anderson, Koella, & Hurd, 1999; Cator, Lynch, Read, & Thomas, 2012; Cator, Lynch, Thomas, & Read, 2014; Cator et al., 2013, 2015; Hurd, 2003; Koella, Rieu, & Paul, 2002; Koella, Sørensen, & Anderson, 1998; Lefèvre & Thomas, 2008; Smallegange et al., 2013; Wekesa, Copeland, & Mwangi, 1992), many questions remain about the extent of such changes in natural vector–parasite combinations and the robustness of the phenomena across environmental conditions (Cornet, Nicot, Rivero, & Gandon, 2013; Vantaux et al., 2015) and whether malaria parasites can manipulate mosquito host choice in ways that enhance parasite transmission toward suitable hosts and/or reduce mosquito attraction to unsuitable hosts (i.e., specific manipulation) (Nguyen et al., 2017).

There is a reason to think that both parasite and host genetics should be selected upon to shape these phenotypes. The altered patterns in feeding behavior observed in malaria‐infected mosquitoes have been empirically demonstrated to have negative impacts on mosquito fitness (Anderson, Knols, & Koella, 2000; Ohm et al., 2016). This suggests that there is selection for both the parasite to alter mosquito behavior and the vector to resist being manipulated (Daoust et al., 2015). Historically, there has been a large emphasis on identifying specific parasite traits that in isolation lead to altered mosquito behavioral phenotypes. Recent work suggests that some components of manipulation may relate to the mosquito's own immune response (Cator et al., 2013, 2015) and that the transmission phenotype observed is likely dependent on the genotype and condition of the vector, as well as the parasite (Cator et al., 2015). How these phenotypes can vary with the environment (e.g., mosquito age or vector density) is unknown and is critical for our understanding of how they affect transmission.

## 4 THE EXTRINSIC INCUBATION PERIOD (*n*)

Natural selection will theoretically favor a developmental schedule for each parasite stage which maximizes transmission between successive hosts (Poulin, 2007). Once in the insect vector, a major challenge facing the parasite is to reach its infective stage before the insect takes its last blood meal. The extrinsic incubation period (EIP) is the duration of the parasite's development within the mosquito that starts with the ingestion of infective malaria parasites, gametocytes, in a blood meal and ends with the sporozoite invasion of the salivary glands when the mosquito becomes infectious (Box 1). For many mosquito–*Plasmodium* associations, this period is as long as the insect vector's average lifespan (Charlwood et al., 1997; Killeen, Mckenzie, Foy, Peter, & Beier, 2000). *Plasmodium falciparum,* for example, has an extremely variable EIP, but generally ranges from 10 to 14 days in high‐transmission settings (WHO, 1975). The question of why this period is so long relative to the vector lifespan has been discussed elsewhere (Cohuet, Harris, Robert, & Fontenille, 2010; Koella, 1999; Ohm et al., 2016; Paul, Ariey, & Robert, 2003).

Both mosquito and parasite are ectothermic, and the impact of temperature on the rate of sporogonic development has long been recognized (Boyd, 1949; Detinova, 1963; Murdock, Paaijmans, Cox‐foster, Read, & Thomas, 2012). In general, warming temperatures speed up parasite development, although above a certain threshold (30°C in *P. falciparum*), this can reduce infection level (Noden, Kent, & Beier, 1995; Okech et al., 2004). Evidence that EIP can vary in response to other environmental factors is limited. *Plasmodium falciparum* EIP can be modified by the quantity of food received by *An. stephensi* larvae (Shapiro et al., 2016) or by the source of plant sugar taken by adult *Anopheles coluzzii* (Hien et al., 2016). Of particular interest would be to test the extent to which malaria parasites can plastically speed up their EIP when their transmission potential is compromised by the imminent death of their vector. Such condition‐dependent developmental strategies, described in other parasite species (Donnell & Hunter, 2009; Poulin, 2007) and in blood‐stage malaria parasites (Mideo & Reece, 2012), deserve consideration in infected mosquitoes. Besides mosquito age, other environmental factors, including exposure to insecticides (Viana, Hughes, Matthiopoulos, Ranson, & Ferguson, 2016) or presence of other parasite species/genotypes (Blanford et al., 2005; Lorenz & Koella, 2012), are associated with mosquito survival and could induce an adaptive plastic shift in parasite EIP. Similar to within‐vector conditions, the extent to which parasite and/or mosquito genetic variation can influence EIP merits exploration.

At the interspecific level, some studies suggest that parasites may adapt to vector lifespan, as demonstrated by *Plasmodium* species with shorter EIPs associating with shorter lived vectors, such as *Plasmodium mexicanum* that is vectored by short‐lived sandflies. Only about 2% of sandflies capable of transmitting *P. mexicanum* live long enough to take a second blood meal (Fialho & Schall, 1995). Compared to other *Plasmodium* species, *P. mexicanum* has a rapid development time that ensures transmission despite the vector's high mortality, which is likely an evolved response.

At the intraspecific level, there has been no study on the influence of parasite and/or mosquito genetics on EIP duration. A recent study investigating the evolutionary potential of dengue virus EIP in *Aedes aegypti* demonstrated that genetic variation among a range of mosquito genetic lines can modulate the length of EIP (Ye et al., 2016). Because vectorial capacity is highly sensitive to changes in EIP, it becomes urgent to investigate the evolutionary potential of EIP in malaria parasites using family‐based breeding (Ye et al., 2016) and/or experimental evolution design (Nidelet, Koella, & Kaltz, 2009).

## 5 MOSQUITO LONGEVITY (*p*) AND OTHER DAMAGES INFLICTED TO THE MOSQUITO

Whether malaria parasites cause fitness costs to their mosquito hosts has received much attention and has long been disputed (Ferguson & Read, 2002b; Hurd, 2009; Vézilier, Nicot, Gandon, & Rivero, 2012). Given the traumatic nature of the sporogonic development (ookinetes and sporozoites perforate the midgut and salivary gland, respectively, Box 1), some degree of virulence (i.e., parasite‐induced fitness cost) might be expected. Malaria infection has been found to increase susceptibility to harmful bacterial and viral infections (Maier, Becker‐Feldman, & Seitz, 1987; Rodrigues, Brayner, Alves, Dixit, & Barillas‐Mury, 2010; Vaughan & Turell, 1996), decrease host energetic reserves (Hurd, Hogg, & Renshaw, 1995; Liu, Dong, Huang, Rasgon, & Agre, 2013; Mack, Samuels, & Vanderberg, 1979a,b), increase sugar intake requirements (Zhao et al., 2012), and decrease flight performance (Schiefer, Ward, & Eldridge, 1977). Furthermore, mounting an immune response to the parasites alone is costly (Ahmed & Hurd, 2006; Blandin, Marois, & Levashina, 2008; Cirimotich et al., 2010). Finally, increased mosquito biting rate induced by sporozoites (see above) can increase feeding‐associated mosquito mortality in the field (Anderson et al., 2000). Together, these mechanisms could negatively impact mosquito longevity and fecundity.

Although both mosquito and parasite could gain fitness benefits from longer vector survival, overall, there seem to be negative effects of infection on mosquito longevity, especially in specific conditions reflecting what occurs in nature, such as nutritional, predation, insecticide, or hydric stress (Aboagye‐Antwi et al., 2010; Alout, Yameogo, et al., 2014; Ferguson, Mackinnon, Chan, & Read, 2003; Lalubin, Delédevant, Glaizot, & Christe, 2014; Roux et al., 2015; Sangare et al., 2014). Furthermore, studies showed that mosquito mortality is influenced by parasite density with heavily infected mosquitoes exhibiting reduced lifespan compared to lightly infected individuals (Dawes et al., 2009; Ferguson et al., 2003; Klein, Harrison, Grove, Dixon, & Andre, 1986; Pollitt et al., 2013). Theory suggests that the optimal level of parasite virulence on mosquito longevity should be stage‐dependent. The parasite should first exhibit a low level of virulence during parasite development to prevent the death of both partners. Once the development is completed and sporozoites are in the salivary glands, parasite genotypes able to increase the biting rate of their mosquito vector could be favored (Koella, 1999; Schwartz & Koella, 2001). Consistent with these predictions, some studies reported greater survivorship in infected than in uninfected mosquitoes during the oocyst infection phase and the opposite when sporozoites have reached maturity (Anderson et al., 2000; Lyimo & Koella, 1992; Roux et al., 2015). Whether the increased survivorship observed in infected individuals during oocyst growth resulted from an active manipulation of the parasite or reflects a compensatory response of the mosquitoes to energy depletion remains unknown. Investigating the importance of parasite genetic variability and interactions with mosquito strain would also deserve consideration. In the *P. chabaudi—An. stephensi* model, there is evidence that different parasite genotypes vary in their effects on mosquito survival and fecundity (Ferguson & Read, 2002a; Ferguson et al., 2003). There also is evidence that some mosquito strains can suffer higher cost of infection by a given parasite genotype than others (Vézilier et al., 2012). Future studies are required to test whether *Plasmodium* genotype by mosquito genotype interactions impact mosquito longevity and fecundity.

## 6 POSSIBLE ASSOCIATIONS AND TRADE‐OFFS AMONG TRANSMISSION TRAITS

**Figure 1:**
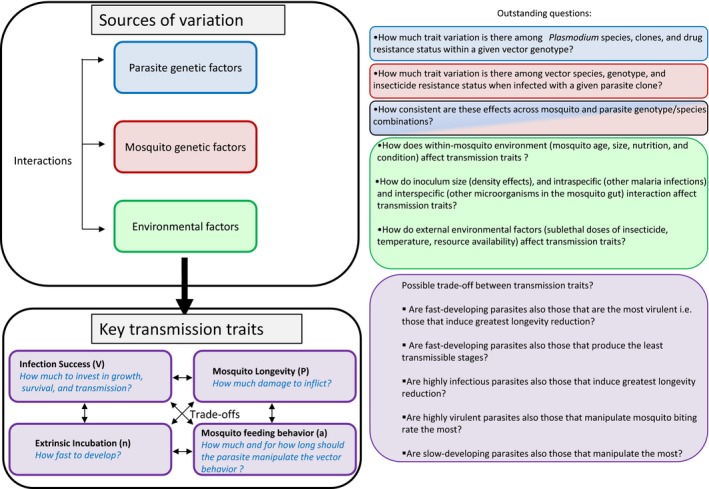
How genetic and environmental factors contribute to variability in extrinsic incubation period, parasite manipulation, infection success, and mosquito longevity and fecundity, remain to be discovered

It is important to remember that it is the emergent properties of a given set of competence, biting rate, EIP, and survival values that determine transmission and that these parameters do not operate in isolation (Figure 1). For example, *Plasmodium* may modify resource allocation of their insect vectors in a way that changes the optimum trade‐off between reproduction and longevity, which, in turn, could favor either longer or similar vector survivorship than uninfected counterparts (Hurd, 2001, 2003, 2009). In a study using avian malaria and allowing mosquitoes to lay their eggs, infected mosquitoes were less fecund but lived longer than uninfected counterparts (Vézilier et al., 2012). This emphasizes the need to concomitantly quantify mosquito longevity and fecundity, which is rarely performed in studies on mosquito–parasite interactions. Finally, there is, to our knowledge, no study that investigated the effect of malaria infection on both mosquito longevity and fecundity over multiple gonotrophic cycles.

Beside the existing links between mosquito infection, fecundity, and longevity, an intriguing possibility is that EIP, the parasite's ability to manipulate mosquito biting rate, and mosquito survival are also correlated. For example, reduced longevity in infected mosquitoes or long parasite development duration will limit the time period for parasite transmission, but this could be compensated by increased mosquito biting rate (Koella, 1999, 2005). In turn, increased biting rate can also increase the probability of mosquito mortality (Anderson et al., 2000). Similarly, the reduction in transmission opportunities due to long parasite development duration could be compensated by increased mosquito lifespan. In other words, fast‐developing parasites might also be those that induce high level of virulence in their mosquito hosts. A recent study using dengue virus‐infected *A. aegypti* revealed that mosquito family lines allowing fast EIP were also those that died faster supporting the existence of a genetic trade‐off between mosquito lifespan and EIP (Ye et al., 2016). To explore these trade‐offs, future work should concomitantly quantify multiple mosquito traits.

## 7 KEY STEPS TO APPLIED VALUE

Understanding how transmission traits of malaria parasites are shaped by the mosquitoes that vector them can inform our approach to disease control. Frontline vector‐borne disease prevention tools such as insecticide‐treated bednets and indoor residual spraying rely on reducing mosquito contact rates with human hosts and reducing vector survival. Reduced vector survival has the benefits of decreasing mosquito abundance, the number of bites a mosquito can take over the course of its lifetime, and the probability that mosquitoes survive past the parasite's development time (Bhatt et al., 2015; Brady et al., 2016; Smith & McKenzie, 2004). These effects likely shape the selective environment for parasites within the vector. Whether parasites can respond to interventions by evolving shorter EIPs or other heritable extended phenotypes that lengthen mosquito survival or change vector behavior merit further investigation.

Human interventions often have evolutionary consequences. For example, it is well known that the use of fast‐kill insecticides selects for rapid insecticide resistance, but the evolutionary and epidemiological impact of evolved resistance traits in vectors on transmission traits of parasites is still not well understood (Alout, Djègbè, et al., 2014; Alout, Yameogo, et al., 2014; Rivero, Vezilier, Weill, Read, & Gandon, 2010). More work evaluating the consequences of insecticide‐resistant mosquitoes on parasite transmission traits will help determine how changing vector traits can influence traits of their co‐evolved parasites. In addition to physiological resistance, how mosquito behavioral resistance in response to LLINs and IRS affects parasite transmission traits is unclear. For example, some studies show that *Anopheles* mosquitoes can shift their host‐feeding behavior from night‐biting to day‐biting following bed net introduction (Moiroux et al., 2012). As diel rhythm shapes mosquito immune responses (Murdock et al., 2013; Rund, Hou, Ward, Collins, & Duffield, 2011; Rund, O'Donnell, Gentile, & Reece, 2016), day‐biting may also alter parasite infection prevalence and intensity. Finally, for interventions not yet deployed, such as late‐life‐acting insecticides or genetically modified mosquitoes, differences in the within‐vector environment parasites experience will also provide potentially different selective forces. Whether parasites can evolve or plastically change transmission traits in response to these interventions needs to be evaluated if we are to responsibly deploy these technologies and prepare for possible evolutionary responses.

While all of these interventions are primarily aimed at and assessed by measuring vector traits, they may have important consequences for parasite evolution. Central to understanding how variation in parasite traits will ultimately influence our approach to control is quantifying how the transmission traits identified in the vectorial capacity equation vary by vector and parasite genotypes, and the plasticity of these traits in the face of selection. Any characterization of these effects should include an estimation of trait heritability across parasite generations, within‐host environments, and external environments. Central to this will be measuring the responses of multiple traits to the within‐vector environment to determine how trade‐offs between them may constrain evolution and dictate parasite transmission.

Finally, because findings on unnatural mosquito–*Plasmodium* associations do not always reflect natural interactions (Aguilar, Dong, Warr, & Dimopoulos, 2005; Boëte, 2005; Cohuet et al., 2006; Dong et al., 2006; Tripet, Aboagye‐Antwi, & Hurd, 2008; Vantaux et al., 2015), it will be essential to follow‐up the discoveries in laboratory model systems such as *P. berghei*—*An. stephensi* or *An. gambiae* with laboratory and field studies on natural parasite–mosquito combinations.
